# Editorial: Endocrinological factors for autism: prenatal biomarkers, early diagnosis and symptom treatment

**DOI:** 10.3389/fendo.2023.1298381

**Published:** 2023-11-09

**Authors:** Yujie Liang, Liyan Zhong, Jianping Lu, Paul Yao

**Affiliations:** ^1^ Department of Child and Adolescent Psychiatry, Shenzhen Kangning Hospital, Shenzhen, China; ^2^ Hainan Women and Children’s Medical Center, Hainan Medical University, Haikou, China

**Keywords:** autism, risk factor, endocrine, hormones, prenatal exposure

The main purpose of the editorial is to summarize the contents of a Research Topic: *Endocrinological Factors for Autism*.

Autism Spectrum Disorder (ASD) is a neurodevelopmental disorder characterized with deficits in social-emotional interactive responses in multiple settings, non-verbal communication behaviors, as well as fixed and stereotyped patterns of behavior or interests. Over the past two decades, the prevalence of ASD has seen a substantial increase, leading to significant impacts on the well-being of affected children and placing a substantial burden on society. Consequently, it has obtained widespread concern and attention from both the public and the research community. It has been shown that ASD may result from the interaction of susceptible genes, immune activation, perinatal disorders, and environmental factors, but its specific etiology and pathogenesis remain unclear, and the current treatment strategies rely on training and special education. An in-depth understanding of the possible environmental risk factors associated with ASD development in children can provide potential insights into early diagnosis, prevention, and early intervention for this disease. In recent studies, researchers have identified numerous potential endocrinological factors that may play a role in ASD development. These factors encompass various hormones such as progestin, androgens, and estrogens (1–4), as well as maternal diabetes (5–7). They have been observed to either elicit autism-like behaviors in rodent models or show associations with the development of ASD in epidemiological research.

Tsompanidis et al. provided a concise overview of the relationship between prenatal androgen exposure and ASD development, and gender-specific behaviors in ASD individuals. It also introduced maternal immune activation-mediated ASD development, which shows a significant sex difference in offspring, thus providing a novel research approach on sex bias in ASD. Research has demonstrated that males exhibit around 4 times the prevalence of ASD symptoms compared to females (8). Tsompanidis et al. focused on the differential gene enrichment that is X-linked in male-preferred placental genes, elucidating the mechanism by which steroid hormones interact with the genetic liability for autism. Physiological measures of early infancy and neurodevelopmental follow-up in the first 2 years of life were utilized in a prospective enrichment cohort study in Cambridge, UK. The results revealed that prenatal testosterone levels, as opposed to postnatal levels, are more pertinent for understanding the origins of autism (2, Tsompanidis et al.). In addition, both gestational hypertensive disorders and polycystic ovary syndrome are associated with ASD, as both cases have hyperandrogenism, and the potential effects on children’s communication and social skills are sex–specific, indicating that maternal testosterone may mediate later autistic development (10). These studies suggest that abnormal testosterone exposure and/or hormonal imbalance may contribute to the male bias of ASD.

Soyer–Gobillard et al. reviewed the effects of neurodevelopmental and psychiatric disorders in children with intrauterine exposure to diethylstilbestrol (DES), providing evidence that *in utero* exposure to DES and other synthetic estrogens can cause psychiatric disorders. Also, it has been reported that endocrinological disorders are associated with gastrointestinal (GI) symptoms during ASD development. Animal experiments have also suggested that maternal diabetes triggers increased intestinal permeability, altered microbiota, and the subsequent development of autism–like behaviors in rodent offspring (7). Further investigation demonstrated that exposure to progestin 17–hydroxyprogesterone caproate (17–OHPC) causes claudin1 (CLDN1) suppression through epigenetic modifications, leading to GI dysfunction in autism–like mouse offspring (4). The enteric nervous system (ENS), as a potent regulator of intestinal barrier function and intestinal homeostasis, plays a key role in ASD pathogenesis that triggered by environmental factors. However, it remains unclear whether GI dysfunction is a consequence of ASD or a potential cause instead. Here, Wang et al. discussed the current understanding of ENS defects associated with ASD and highlighted the widespread expression of several high–risk genes in the gut related to ASD, providing new perspectives for understanding endocrinological disorders with ASD–related GI dysfunction. In addition, neuroligin (NLGN) has shown significant contribution and important functions in synaptic development, whereas the potential relationship between gestational diabetes and NLGNs in ASD remains unclear. Zhang et al. first identified different expression levels of NLGNs in an autism–like mouse model induced by streptozotocin, and the cryo–electron microscopic structures for human NLGNs were determined, indicating potential different mechanisms of ASD related to NLGN structures.

Recently, there has been an increase in our knowledge of the causative factors of autism. Endocrine factors are one of the possible causal or susceptibility factors for autism. There is mounting evidence to suggest that dysregulations in the endocrine system could be implicated in the pathogenesis of ASD. Understanding the endocrine aspect of autism not only enhances our comprehension of its underlying causes but also opens the door to novel therapeutic strategies. This knowledge can provide the way for the development of interventions aimed at ameliorating the impact of endocrine imbalances, eventually improving the lives of individuals with ASD and their families.

## Author contributions

YL: Writing – original draft. LZ: Writing – original draft. JL: Conceptualization, Writing – review & editing. PY: Conceptualization, Writing – original draft, Writing – review & editing.
